# Parasite resistance and parasite tolerance: insights into transgenerational immune priming in an invertebrate host

**DOI:** 10.1098/rsbl.2022.0018

**Published:** 2022-04-06

**Authors:** Sofia Paraskevopoulou, Sabrina Gattis, Frida Ben-Ami

**Affiliations:** School of Zoology, George S. Wise Faculty of Life Sciences, Tel Aviv University, Tel Aviv, 6997801, Israel

**Keywords:** pathogens, *Daphnia*, *Metschnikowia*, maternal effects, grandmaternal effects, resistance

## Abstract

Parasites impose different selection regimes on their hosts, which respond by increasing their resistance and/or tolerance. Parental challenge with parasites can enhance the immune response of their offspring, a phenomenon documented in invertebrates and termed transgenerational immune priming. We exposed two parental generations of the model organism *Daphnia magna* to the horizontally transmitted parasitic yeast *Metschnikowia bicuspidata* and recorded resistance- and tolerance-related traits in the offspring generation. We hypothesized that parentally primed offspring will increase either their resistance or their tolerance to the parasite. Our susceptibility assays revealed no impact of parental exposure on offspring resistance. Nonetheless, different fitness-related traits, which are indicative of tolerance, were altered. Specifically, maternal priming increased offspring production and decreased survival. Grandmaternal priming positively affected age at first reproduction and negatively affected brood size at first reproduction. Interestingly, both maternal and grandmaternal priming significantly reduced within-host–parasite proliferation. Nevertheless, *Daphnia* primed for two consecutive generations had no competitive advantage in comparison to unprimed ones, implying additive maternal and grandmaternal effects. Our findings do not support evidence of transgenerational immune priming from bacterial infections in the same host species, thus, emphasizing that transgenerational immune responses may not be consistent even within the same host species.

## 1. Introduction

During their lifespan, organisms are exposed to various parasites (including pathogens) that affect numerous phenotypic traits and consequently reduce their fitness [1]. The presence of parasites may enhance the immune response of the challenged individuals or, based on their own immunological experience, the immune response of their offspring—a phenomenon termed transgenerational immune priming (TGIP; [2,3]). Theoretical models predict that TGIP will be favoured when ecological conditions between the host and its parasites are stable over time [4]. In such cases, there is a higher chance that hosts and their offspring encounter the same parasite species, in which case a response via TGIP would probably increase resistance to their parasite (i.e. reduce parasite fitness) and inhibit disease spread [2,3]. Although beneficial, TGIP is not a consistent mechanism, since the evolution of increased resistance to parasites may bear fitness costs for the offspring or their parents [2,3]. Alternatively, hosts can increase their tolerance (i.e. limit the damage caused by a parasite/virulence without affecting parasite fitness) by modifying fitness-related life-history traits [5–7]. Therefore, the evolution of both parasite resistance and parasite tolerance can influence population dynamics and parasite virulence, which poses an important challenge for epidemiological theory [8].

Cyclically parthenogenetic species like the invertebrate *Daphnia magna* offer a conceptual framework to study TGIP and its consequences. Due to their asexual life cycle, genetic and non-genetic effects can be easily disentangled, while their short generation time increases the probability of parents and offspring encountering the same parasites in their environment. From an ecological perspective, *Daphnia* species are key players in aquatic environments, due to their contribution to aquatic trophic webs [9]. Therefore, investigating their immune response and adaptive potentials is critical to better predict disease spread and population dynamics during disease outbreaks [10].

## 2. Material and methods

In this study, we challenged *D. magna* hosts with the exclusively horizontally transmitted parasitic yeast *Metschnikowia bicuspidata* [11,12] for two consecutive generations and assessed their offspring's resistance and tolerance. We hypothesized that primed offspring would be more resistant or, if resistance is costly, more tolerant to the parasite in comparison to unprimed offspring. Specifically, we tested whether primed offspring coped better with a homologous parasite challenge than unprimed ones (the ‘environmental matching' hypothesis). We further tested whether offspring born to mothers previously exposed to the parasite had a reduced fitness, regardless of the offspring treatment (the ‘stress' hypothesis) [13–15].

### (a) Host and parasite genotypes

As hosts, we used two *D. magna* clones: ISR (clone HSS1, Israel, 2015) and NOV (clone NOV7C, Norway, 2014). Individuals from both clones were exposed to an *M. bicuspidata* isolate originating from infected hosts collected from Ammersee Lake, Germany (isolate AMME, Germany, 2008). The parasite was maintained under constant replication using as a host another *D. magna* clonal line, different from the experimental clones.

### (b) Experimental design and phenotypic experiments

Before the initiation of the experiment, animals were acclimated for three generations under a cycle of 16 : 8 L/D at 20°C ± 1°C to minimize maternal effects and allow for a split-brood experimental design (electronic supplementary material).

For each host clone, we took 160 5-day-old female offspring from the first clutch and placed them individually in a jar filled with 20 ml of artificial *Daphnia* medium [16]. This cohort served as the grandmaternal generation (F_0_). Animals were split into two treatments: unprimed (unexposed to the parasite) and primed, by exposing them to 500 spores ml^−1^ of *M. bicuspidata* for 5 days. To prepare the spore vials for the inoculation process, *Daphnia* infected with *M. bicuspidata* were yielded from the original culture, crashed with a plastic sterile pestle and diluted to the concentration of 500 spores ml^−1^. The unexposed treatment received an equal amount of crushed uninfected *Daphnia* as a placebo. On the first day of inoculation, animals were not fed to allow for spore digestion, and medium was not changed during the entire inoculation period. On day 5 post exposure, animals were transferred to jars with fresh medium and thereafter, medium and jars were replaced every third day or when offspring were present. *Scenedesmus* sp. was provided *ad libitum* as food source following an age-structured food intake (electronic supplementary material).

**Figure 1.**
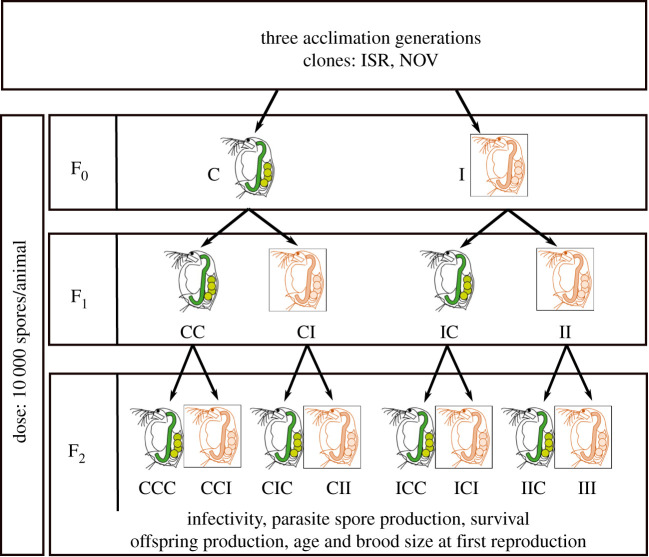
Experimental design to investigate transgenerational effects of immune priming in the *Daphnia magna* – *Metschnikowia bicuspidata* system. ‘C', unprimed; ‘I', primed. F_0_, F_1_ and F_2_ represent the three generations of the TGIP experiment in sequential order, respectively.

Approximately 10 days post exposure, infection was determined under a dissection microscope (Leica M205), and infected individuals of the F_0_ generation were sorted out. First brood offspring from both infected (I) and unprimed (C) F_0_ individuals were allocated again into two treatments: unprimed and primed by the parasite, following the same infection process as in the F_0_ generation. Hence, the F_1_ generation comprised four treatments (CC, F_0_-unprimed/F_1_-unprimed; CI, F_0_-unprimed/F_1_-primed; IC, F_0_-primed/F_1_-unprimed; II, F_0_-primed/F_1_-primed). Following a cross-factorial design, the F_2_ generation was established similarly to the F_0_ and F_1_ generations, and its newborns received either the parasite or the placebo treatment. This resulted in an experimental design of eight treatments (CCC, CCI, CIC, CII, ICC, ICI, IIC, III) for each clone (figure 1). The sequence of letters represents the treatments received in the F_0_, F_1_ and F_2_ generations, respectively, with ‘C' standing for unprimed animals and ‘I' standing for primed animals.

### (c) Data collection and statistical analyses

As a proxy for parasite resistance, we measured infectivity, i.e. the proportion of infected animals in the F_2_ generation. Life-history traits, i.e. age at first reproduction (AFR) and brood size at first reproduction (BSFR), offspring production and survival were recorded as proxies to parasite tolerance. AFR was defined as the day of releasing the first brood from the brood pouch. We excluded from further analysis exposed-but-uninfected animals and males that occurred at low frequencies. All phenotypic traits were recorded upon host death. Dead individuals were crushed with a sterile plastic pestle, and spores were counted twice in 10 ul of water, on a Neubauer improved counting chamber under a phase-contrast microscope (Leica DM2500), as a proxy for parasite fitness.

To compare life-history traits among the F_2_ generation, we applied generalized linear models to all traits (except spore production) due to deviations from normality and homoscedasticity [17]. Thus, error distributions were assigned to each trait by fitting the ‘fitdist' function (‘fitdistrplus' package, [18]). Offspring production and BSFR were modelled with a negative binomial distribution to account for over-dispersion, while survival and AFR were modelled with a gamma distribution. Infectivity, as a binary variable, was analysed using binary logistic regression. Spore production data met the criteria for linear regression modelling.

Host clone (ISR, NOV), infection (F_2_ generation treatments: C, I), maternal/F_1_ priming (C, I) and grandmaternal/F_0_ priming (C, I) were modelled as two-level fixed effects. The most parsimonious model, i.e. the one with the smallest corrected Akaike information criterion (AICc) value, was selected with the ‘dredge' function of the ‘MuMIN' package [19]. Statistical significances for each variable included in the model were obtained with the function analysis of variance (model, type = 2). *Post hoc* comparisons were computed using the ‘emmeans' package [20]. All statistical analyses were performed using R v. 4.0.4, while for visualization, the package ‘ggplot2' was used [21].

## 3. Results

**Figure 2.**
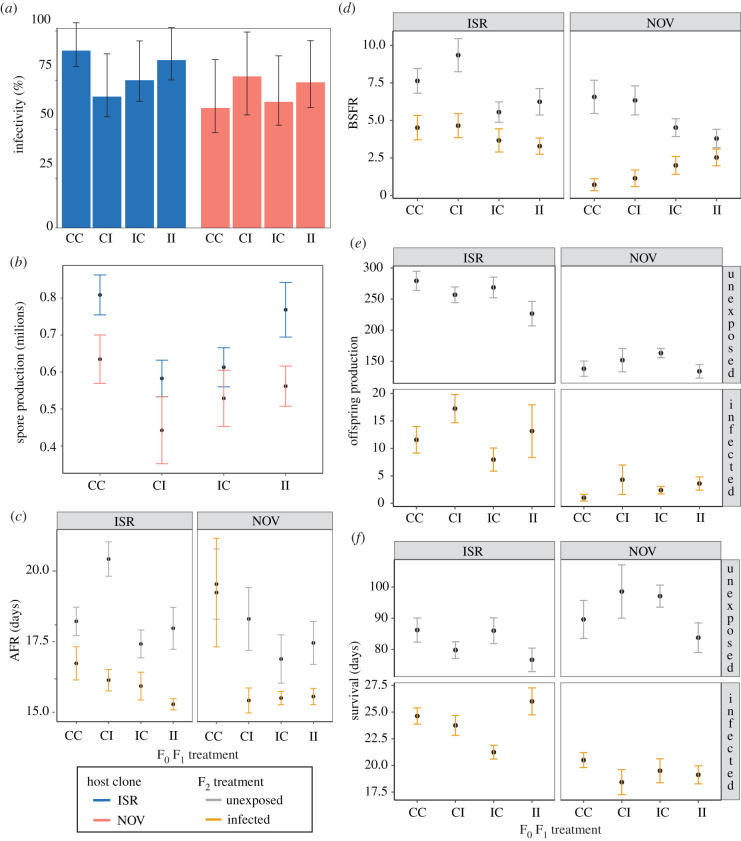
(*a*) Infectivity, (*b*) mean parasite spore production, (*c*) AFR, (*d*) BSFR, (*e*) offspring production and (*f*) survival per clone, F_0_, F_1_ and F_2_ treatments. CC: F_0_-unprimed/F_1_-unprimed; CI: F_0_-unprimed/F_1_-primed; IC: F_0_-primed/F_1_-unprimed; II: F_0_-primed/F_1_-primed. In (*a*), error bars represent Wilson Score 95% CIs. In (*b–f*), error bars indicate standard errors.

Parasite resistance, estimated via infectivity, was unaffected by maternal or grandmaternal priming (table 1 and figure 2*a*). In comparison to unprimed animals, spore production was significantly reduced in both grandmaternally and maternally primed animals (*post hoc:* CC versus IC, *p* = 0.048; CC versus CI, *p* = 0.009; tables 1 and 2 and figure 2*b*). Spore accumulation, however, was not significantly different between unprimed animals and animals whose mothers and grandmothers were both primed (*post hoc:* CC versus II, *p* = 0.78).

**Table 1.** Generalized and general linear models of the effects of grandmaternal/F_0_ priming, maternal/F_1_ priming, F_2_ treatment, host clone and their interactions on various fitness-related traits. The model with the smallest corrected Akaike information criterion (AICc) value is presented. LR, likelihood ratio. Bold typeface indicates significant effects.

trait type	predicted variable	independent variables	d.f.	LR	*p*-value
resistance trait	infectivity	host clone	1	3.21	0.073
tolerance traits	spore production	host clone	1	10.68	**0.001**
		F_0_ priming	1	0.19	0.667
		F_1_ priming	1	0.93	0.337
		F_0_ priming × F_1_ priming	1	12.16	**<0.001**
	age at first reproduction	F_0_ priming	1	17.42	**<0.001**
		F_1_ priming	1	0.77	0.381
		F_2_ treatment	1	34.58	**<0.001**
		F_1_ priming × F_2_ treatment	1	3.277	0.070
	brood size at first reproduction	host clone	1	29.92	**<0.001**
		F_0_ priming	1	8.94	**0.003**
		F_2_ treatment	1	70.58	**<0.001**
		F_0_ priming × F_2_ treatment	1	6.59	**0.010**
		host clone × F_0_ priming	1	2.63	0.105
		host clone × F_2_ treatment	1	9.23	**0.002**
		host clone × F_0_ priming × F_2_ treatment	1	10.82	**0.001**
	survival	host clone	1	3.32	0.068
		F_1_ priming	1	5.25	**0.022**
		F_2_ treatment	1	2584.05	**<0.001**
		host clone × F_2_ treatment	1	33.00	**<0.001**
	offspring production	host clone	1	90.49	**<0.001**
		F_1_ priming	1	1.91	0.167
		F_2_ treatment	1	986.54	**<0.001**
		host clone × F_2_ treatment	1	25.63	**<0.001**
		F_1_ priming × F_2_ treatment	1	12.14	**<0.001**

**Table 2.** Summary of the impact of parental effects for each fitness-related trait.

trait type	significant parental effect	fitness trait	F_2_ generation treatment	priming effect
resistance trait	none	infectivity^a^	infected	no effect
tolerance traits	grandmaternal (F_0_)	age at first reproduction	unexposed infected	positive
		brood size at first reproduction	unexposed infected	negative positive for NOV no effect for ISR
	maternal (F_1_)	offspring production	unexposed infected	no effect positive
		survival	unexposed infected	negative
	grandmaternal (F_0_) × maternal (F_1_)	spore production^a^	infected	positive/additive

^a^These traits apply only to infected animals.

Infected animals reproduced earlier (*p* < 0.001), produced less offspring (*p* < 0.001) and survived for a shorter period than unexposed ones (*p* < 0.001; table 1). Grandmaternally primed *Daphnia* reproduced earlier than unprimed ones (*p* < 0.001; tables 1 and 2 and figure 2*c*). Grandmaternal priming differently affected BSFR in the two clones. On the one hand, animals from both clones that had not been infected by the parasite experienced reduced BSFR after grandmaternal priming (*post hoc*: clone = ISR, F_2_ = C, F_0_-C versus F_0_-I, *p* = 0.006; clone = NOV, F_2_ = C, F_0_-C versus F_0_-I, *p* = 0.008). On the other hand, BSFR was higher in grandmaternally primed animals from the NOV clone, which became infected in the F_2_ generation (*post hoc*: clone = NOV, F_2_ = I, F_0_-C versus F_0_-I, *p* = 0.002; tables 1 and 2 and figure 2*d*). Maternal priming increased offspring production in infected animals (*p* < 0.001; tables 1 and 2 and figure 2*e*), while survival in general decreased (*p* = 0.022, tables 1 and 2 and figure 2*f*).

The NOV clone was marginally more resistant (*p* = 0.07; table 1), more tolerant to parasite proliferation (*p* < 0.001; table 1) and produced fewer offspring than the ISR clone (*p* < 0.001; table 1). The NOV clone also survived longer in a parasite-free environment, albeit infection severely reduced its lifespan in comparison to the ISR clone (figure 2*f*).

## 4. Discussion

Parentally primed animals were more tolerant to infection in comparison to unprimed ones. They were not, however, more resistant to infection, likely due to its costs. Furthermore, parentally primed animals that were unexposed in the F_2_ generation exhibited immune triggering-related costs in multiple life-history traits.

Grandmaternal priming significantly affected early life-history traits such as AFR and BSFR, suggesting that parental priming may span multiple generations. Both grandmaternal priming and infection reduced AFR. One possible explanation might be fecundity compensation [22–24], whereby infected hosts shift their resource allocation towards early reproduction to increase offspring production before the parasite begins to exploit host resources [25,26]. Early reproduction often comes at the cost of longevity, potentially reducing offspring lifetime fitness [27]. While such a trade-off was noticeable for infected offspring whose survival was shorter than unexposed ones, it was not evident between grandmaternally primed and unprimed animals. Thus, it is unlikely that grandmaternally primed animals completed their development earlier than unprimed ones, hence providing them a fitness advantage [28]. Grandmaternal priming reduced BSFR in unexposed animals, which suggests that immune triggering may bear some costs even two generations after the threat of parasites had been removed. For infected animals, grandmaternal priming increased BSFR, thus providing a competitive advantage for these animals when becoming infected. The increase in BSFR was clone-specific, implying that priming effects may have a genetic basis. Brood size and offspring size typically trade off in response to changes in offspring investment [29]. Nevertheless, this was not consistent, because exposure of parental *Daphnia* generations to fungicides demonstrated that more offspring can be produced without compensating for the cost of size [30].

Late life-history traits such as survival and total fecundity were primarily affected by maternal treatment, thus emphasizing the importance of maternal priming for offspring fitness. Maternal priming positively affected offspring production in infected animals, supporting the prediction of the ‘environmental matching' hypothesis that matching environments provide a fitness advantage to the offspring even when the environment being matched is stressful [31,32]. Our results contradict findings in other daphniids, where offspring born to infected mothers suffered reduced fecundity, possibly as a by-product of stress [33]. In contrast to offspring production, survival was negatively affected by maternal priming. Such a ‘stress' response indicates again that triggering the immune system may bear fitness costs to the offspring generation. Likely, this trade-off between survival and fecundity suggests that animals allocate more resources towards reproduction than towards survival.

Interestingly, spore accumulation was affected by both maternal and grandmaternal priming. Although one primed generation (F_0_ or F_1_) was sufficient to reduce spore accumulation, two consecutive primed generations were not, thus indicating additive maternal and grandmaternal effects. Parental effects can sometimes be indirect, resulting in a mixture of seemingly adaptive and maladaptive effects [34]. To this extent, it remains to be determined whether parental challenge endures adaptive immune priming in our system.

We observed clonal variation in the majority of phenotypic traits. In the absence of the parasite, the survival of clone ISR was shorter than clone NOV, whereas in the presence of the parasite, the survival of clone ISR was longer, and it accumulated more spores than clone NOV. Therefore, faster-developing clones may favour faster exploitation by the parasite. Such trade-offs in cue integration may reflect genotype-by-genotype (GxG) interactions or be related to the environments where these clones had evolved. However, since the genetic variability of *M. bicuspidata* is limited [35,36], any conclusions regarding GXG interactions are premature.

Contrary to our expectations, the susceptibility assays did not reveal a significant effect of parental priming on offspring resistance to infection. Our findings are consistent with studies of the *Daphnia dentifera*–*M. bicuspidata* system and other invertebrates (e.g. the mealworm *Tenebrio molitor*) challenged with fungi, whereby offspring of primed mothers were not more resistant to homologous challenges [33,37]. Our results contradict, however, previous findings from the *Daphnia–Pasteuria* system, in which mothers primed against Gram-positive bacteria decreased their offspring's susceptibility to homologous species challenges [4,26,38,39]. By forming endospores, *Pasteuria ramosa* is the most persistent pathogen in the external environment of *D. magna*. Hence, a differential TGIP induction between fungi and bacteria may imply that the latter has been an important selective force for the evolution of immune priming in *D. magna*. An alternative explanation for our results relies on possible mediation of TGIP by within-generation developmental plasticity, since a part of juvenile development (until day 5) took place in a parasite-free environment. Similarly, conflicting results have been reported regarding thermal transgenerational effects when part of the juvenile life was spent in the maternal treatment [40,41]. Finally, the absence of resistance might be related to dose effects and the predictability of infections [26,42]. Maternal challenge with smaller parasite doses at more frequent intervals may shed light on whether TGIP can induce changes in offspring pathogen resistance.

Due to redundancy of underlying (immune) processes, resistance and tolerance can be independent, positively correlated or traded off against each other [43,44]. Although not significant, we captured a trade-off between resistance and tolerance. The Norwegian clone (NOV) that was marginally more resistant to infection exhibited less tolerance by producing fewer offspring and surviving for a shorter period than the ISR clone, and *vice versa*. Even when the short-term benefits of resistance and tolerance are the same for the host, their evolutionary outcomes may differ [45]. Resistance mechanisms directly inhibit infection, thereby reducing parasite fitness. On the contrary, tolerance mechanisms may increase parasite prevalence by allowing infected hosts to live longer, positively reflecting on their fitness [45]. Thus, a negative coupling between resistance and tolerance might indicate host–parasite coevolution [46].

While parental effects on fitness-related traits were detected, whether such shifts are adaptive necessitates further exploration. Transgenerational effects on fitness-related traits, however, may impose an important challenge for epidemiological theory, since standard ‘susceptible–infected–recovered' models usually underestimate their contribution, and thus fail to capture the whole spectrum of disease dynamics and spread. Importantly, a discrepancy with previous findings described for the *Daphnia–Pasteuria* system implies that the priming mechanism is not consistent even within the same host species. Therefore, epidemiological models should be used with caution if developed for another host–parasite system. Elucidating the molecular basis underlying such trait shifts by exploring gene expression patterns or epigenetic changes that are altered between primed and unprimed offspring will enhance our understanding of the induction of TGIP. This, in return, would potentially shed light on the biochemical pathways that are involved in TGIP and the host resources that the parasite exploits during fungal infections.

## Data Availability

All data are available online as the electronic supplementary material and deposited in Dryad Digital Repository: https://doi.org/10.5061/dryad.c59zw3r98 [47]. The data are provided in electronic supplementary material [48].
